# Strengthening primary healthcare through community involvement in Cross River State, Nigeria: a descriptive study

**DOI:** 10.11604/pamj.2014.17.221.2504

**Published:** 2014-03-21

**Authors:** Hilary Adie, Thomas Igbang, Akaninyene Otu, Ekanem Braide, Okpok Okon, Edet Ikpi, Charles Joseph, Alexander Desousa, Johannes Sommerfeld

**Affiliations:** 1 Ministry of Health, Calabar, Nigeria; 2 Department of Internal Medicine, University of Calabar Teaching Hospital, Calabar, Nigeria; 3 Federal University, Lafia, Nasarawa State, Nigeria; 4 Zoology/Environment Biology Department University of Calabar, Calabar, Nigeria; 5 University of Calabar Teaching Hospital, Calabar, Nigeria; 6 Department of Sociology, University of Calabar, Nigeria; 7 World Health Organization, Geneva, Switzerland

**Keywords:** Primary Health Care, Community directed Interventions, key informant interviews, focus group discussions, Cross River State

## Abstract

**Introduction:**

In preparation for implementation of primary healthcare (PHC) services in Cross River State, a study to identify perceptions of communities and health systems concerning such interventions was conducted.

**Methods:**

Existing PHC practices were documented through observation and document reviews, including facility checklists at frontline levels. Perceptions of consumers and providers on PHC were elucidated through 32 Focus Group Discussions (FGDs) and 78 semi-structured questionnaires.

**Results:**

There was some level of implementation of the Nigerian PHC policy in the study districts. However, this policy emphasized curative instead of preventive services. Private partners perceived healthcare programmes as largely donor driven with poor release of allocations for health by government.

**Conclusion:**

Both providers and consumers presented similar perceptions on the current PHC implementation and similar perspectives on services to be prioritized. These common views together with their on-going participatory experience are important platforms for strengthening community participation in the delivery of PHC.

## Introduction

National Health systems in many African countries are weak, under staffed, under financed, and decimated by migration and illness. Service provision is also skewed heavily in favour of urban populations [1]. As in other states in Nigeria, successive governments of Cross River State have strived to improve the health of Nigerians, through a series of national development plans and annual budgets. There has been a major reversal of health gains over the past decade: childhood immunization plummeted and has not improved much in the last decade. In consequence, the country is largely not on course to achieve the Millennium Development Goals by 2015 [2].

The infant mortality rate for Nigeria has deteriorated from a rank of 265 in 1970 to 186 in 2008 [3]. Nigeria has consistently under performed in all health and development indices. The life expectancy at birth declined to 44 years in 2009, According to the NDHS 2008 report, health indices from the Southern part of Nigeria are better than those from the North [2]. However, Cross River state which is located in the South-South geo-political zone of Nigeria has poor indices that are comparable to that of the Northern states. Nevertheless, some health care interventions have been resilient to the general decadence in the system. Some have even improved in the last decade and these include certain nutritional programs and child health services such as treatment of diarrhoea and ARI symptoms. These resilient programmes have incorporated some basic aspects of the Alma Ata concept of PHC and have taken community participation as a key strategy for implementation.

This study was aimed at understanding the context of PHC implementation, including consumers and providers perspectives on PHC priorities; it identified opportunities that can be adequately utilized to strengthen primary health care using CDI. The existing government policy regarding PHC was documented, the level of implementation of existing PHC policies at the district and community levels was assessed and priority health issues within the health system and at the community level identified. Also identified were intervention strategies for primary health care delivery at the district and community levels and partners and stakeholders involved in the delivery of PHC interventions. Human and material resources available for PHC activities were ascertained.

The perception and attitude of the community and health care providers about policy and health services was assessed. The cost of health services, community participation and existing PHC interventions were also considered. Other issues included the role of gender, minority groups, interest groups and other socio political organizations in PHC implementation. The challenges, opportunities and synergies in the delivery of PHC interventions were determined. The number, types and extent of community contribution and participation in current and previous health and development activities were also considered.

## Methods

**National level**: At the national level, the relevant Key Informant Interview (KII) guide was administered on the Director of Public Health in the Federal Ministry of Health, the Health Advisor of DFID, the Director of Disease Control at the World Health Organisation, the Project Officer Health of European Union (EU) and the Representative of the Executive Director of the National Primary Health Care Development Agency. Programme annual plans/reports where available were also collected from these organisations for document review. This was purposively done in line with the study protocol and these respondents were considered to be competent sources of information for the research.

**State level**: At the state level, four partners Canadian International Development Agency (CIDA), WHO and Global HIV/AIDS Initiative Nigeria (GHAIN) and Tulsi Chrai Foundation (a United States Agency for International Development funded programme) were interviewed.

**LGA level**: At the LGA level, the PHC Directors in the four (4) study LGAs were interviewed using the appropriate Key Informant Interview (KII) guide for that level. In addition 4 health workers involved in the implementation of various disease control programmes were also interviewed in each LGA. As with the national and state, no sampling was required as the interviewees were specified in the study protocol. Documents where available were reviewed and checklists administered.

**FLHF level**: In each of the four (4) study LGAs, two FLHFs were selected at random. This gave a total of eight (8) FLHFs for the study LGAs of Calabar South, Calabar Municipality, Bakassi, and Abi. The officers in charge of the selected FLHFs were interviewed using the appropriate KII FLHF. Available documents were reviewed and checklists administered.

**Community level**: Four (4) communities were selected at random per facility giving a total of 32. The respective village leaders of the 32 communities were interviewed. In addition, 20 Focus group Discussions (FGDs) were conducted for the following groups: adult male, adult female, youth female and youth male per health facility.

**Ethical Clearance**: This was obtained from the Ethical Board of the Department of Clinical Governance, SERVICOM and E-Health, Ministry of Health, Calabar, Cross River State, Nigeria. Ref. No. CRS/MH/CGSE-H/010/VOL.1 of 25th Nov. 2008.

**Statistics**: The interviews were collated and imputed into AtlasTi software for Qualitative Data analyses. The AtlasTi has annotated sub-themes for the input of the information derived from the interview schedule. The comments from the interviews were entered in word and analyzed using Atlas Ti. 6.1.17.

## Results

### Existing Health Policy

The National policy on health has primary health care playing a pivotal role. It covers the eight original elements of primary health care as outlined by the Alma Ata declaration. There are three additional elements to the original eight, these include; community eye care, community mental care, and community dental care. The policy document prescribes inter-sectoral collaboration, community participation, self reliance, equity and integration of health care programmes in line with international standards. While some people in this study perceived PHC as stipulated in the national health policy as adequate, others believed that the absence of a PHC policy was a weakness in the system. “The national PHC policy contains the standard list of PHC activities and so it has no defect. You should remember that the PHC system used the world over was developed by a Nigerian and it has worked for other parts of the world so I believe it is good for Nigeria. There is absolutely nothing wrong with the policy. It is excellent”. **In-depth interview with Health Advisor, DFID Headquarters, Abuja, Nigeria**.

### Level of implementation of PHC

Only immunization services were provided in a comprehensive manner in all health facilities. Difficulties were experienced with the provision of other services. The strengths and weaknesses of the existing system are obvious from the responses *“I am happy that we are doing our best to meet the needs of the people. At least we carry out most of the activities that the primary health care should carry out. However, it has not been easy. We have about 28 creeks in the riverine areas. We lack speed boats and life jackets for mobility and safety. There is no imprest to run the health facilities and programmes. Our weaknesses are many, I cannot list them all. Though our council is new, we are using the temporary site. We pray that things should improve when we finally move to our permanent site.”* **In-depth interview with PHC Director, Bakassi LGA**.

Both providers and consumers perceived the provision of free drugs for women and children as a welcome development as it had improved patronage of the health facility. However, the males were not happy that these facilities were extended to women and children only. Also, the drugs provided did not cater for health needs and stock outs were frequent. *“Government is trying. However, money given to the councillor representing the ward as community leader is not properly utilized. This is the first time any government official is coming to the community to ascertain the health status and challenges of its inhabitants. There is no standard health centre. The health post at Ibeko Omin Omin is the only one serving the entire Bakoko clan. In other words, the clan has one functional health post and a non-functional one at Eko - Odoso AKA hard to reach.”* **FGD with male adults in Bakoko, Calabar Municipality**.

**Priority Health issues:** Health issues were ranked according to priority based on the morbidity and mortality arising from the various diseases. There was a general consensus on the perception of malaria as a major health issue by all stakeholders [4].

**Intervention strategies for PHC:** The interventions covered decreased from the LGA level down to the community level. The existing interventions followed standard internationally acceptable protocols. *“About 90% of the population from my assessment, has benefited from all the PHC interventions, the remaining 10% represent those at the riverine areas which are hard to reach.”* **In-depth interview with first line health officer, Edim Otop, Calabar Municipality**.

**Partners and Stakeholders involved in delivery of PHC:** The State has a number of NGOs, NGDOs and CBOs collaborating with Government at State, Local Government and community levels. The NGDOs made up of United Nations Agencies supported system strengthening and provided logistics, finances and technical support. Most times, drugs and other commodities were also supplied by these agencies. Other local CBOs and NGOs supported the dissemination of information on health services, implementation and advocacy to policy makers.

**Effectiveness of strategies in the implementation of PHC:** The service users“ perception at the community was diverse; some considered the services they received to be effective, while others did not feel so. They were however dissatisfied with the limited supply of commodities and inadequate number of health personnel to address their health needs. The largest cadre of health workers identified was the community health extension workers.

**Financial / Human resources:** FGDs and KII revealed that women wielded a considerable amount of influence in the community. Community school blocks and health centers built were initiated and inaugurated by women. Primary Healthcare was on the concurrent list. At the national level, the department of public health had a budget of 800 million Naira ($5.3 million) which was released to the department *“We had a budget of N800m ($5.3million) in 2009, the money was released fully and we attained more than 95% ($5.023) expenditure.”* In-depth interview with National Director PHC, Abuja.

The study team was however not given a breakdown of the budget and expenditure. EU PRIME, a European Union supported programme, spent 97.4 million Euros. EU PRIME is a programme for promoting routine immunization. EU also spent 1.2 million Euros in the past seven years also on HIV/AIDs, TB and adolescent health. *“We have supported the PRIME programme for 7 years with 97.4 million Euros in the first strategic plan. This was as support to polio eradication through the federal government basket fund. The support was for routine immunization service delivery, management, capacity building, coordination and provision of infrastructure. The essence was to ensure quality routine immunization services in 6 states. This was extended to 17 additional states and FCT bringing it up to 23 states. The support was strategic because service delivery is poor. Immunization brings mother and child to the health facilities and so is a strategic entry point for PHC.”* In-depth interview with Programme Officer Health EU PRIME, Abuja, Nigeria. Canadian International Development Agency “ CIDA****. There was uneven distribution of health resources. While some Health centres were fairly equipped, some lacked basis equipment to work with. *“The building is not fenced thus it is exposed to outsiders, especially mechanics and miscreants who are very many in this area. Part of the building has fallen. The two health posts under this facility are using church verandas, no structures. We need extra rooms for other activities like children's ward, family planning unit, laboratory unit, etc. There has been no light in our environment for five years now because the transformer got bad. We need a generating plant for the health centre. We need forceps for delivery, suction apparatus, oxygen and weighing scales. The solar refrigerator we use to store our vaccines does not function all the time but we are managing it, it is better than nothing. The facility needs beds. The 4 beds presently in use are in such bad shape that patients prefer to lie on the floor.”* In-depth interview with FLHF, Afokang, Calabar South LGA.

Communities in Abi and Bakassi LGAs felt healthcare services were inadequate and unaffordable. Communities were told of upcoming health interventions and their cooperation was sought for by health workers. However, communities were not involved in the overall planning process. Community members saw some of the health interventions as inappropriate. They felt they could have selected better intervention strategies if they had been involved in the planning. *“My people prefer the government clinic but the workers charge too much and the clinic is too far from the village. The council of chiefs rented a house for the new midwives sent to us. We also bought mattress for them. We wrote to the government for the repair of the solar system and water project. We encouraged the youths to clear the surroundings. We are always ready to help when the nurses call on us.”* In depth interview, community leader, Enorisoni, Abi LGA.

### Perception of community, health providers, leaders and partners on policy, health issues, service providers, cost of service, community participation and existing PHC interventions

Health care providers felt the PHC policy did not address all components of PHC. They attributed this to inadequate budgetary provisions for PHC at all levels. They were not satisfied with working conditions because of low salaries and lack of motivation, training and promotion. The cost of services was viewed as affordable. The health workers felt community participation in health care was good but saw the need to engage communities in the decision making process involving their health. There was dissatisfaction with interventions carried out as some were too general and did not address specific needs. The purpose of the interventions in many cases was not known by community members. *“Actually, I need to speak the truth. Most of the work we do is achieved using personal materials and funds. We require facilities such as a nutrition demonstration room which is not available. In fact, I am not satisfied. Personal funds are used to pay for photocopying of documents in a bid to keep records and statistics.”* In depth interview, Nutrition Officer, Bakassi LGA

### Role of gender, minority groups, interest groups and other socio political organisations in PHC implementation

The three LGAs of Calabar Municipality, Calabar South and Bakassi were heterogenous with inhabitants made up of fishermen and farmers from other neighboring states. The ethnic groups found in these areas were Ijaws, Ibos, Ibibios and Camerounians. However, there was a meeting point for harmonious relations through the churches, social clubs and community development associations where all the groups met for development work within the community. The minority groups of Ijaws, Ibos, Ibibios and Camerounians did not perceive themselves as such since they shared the same Christian belief. The women in all the communities visited played very significant roles in health care delivery.

### Number, the types, and the extent of community contribution and participation in current and previous health and development activities

Community members were involved in various health and development activities in the community to ensure the well being of the citizenry. The activities undertaken at any point in time depended on the existing needs at a particular point in time. Community members were engaged in self help and it was customary to find such gestures with proper mobilization. Community members had on their own organized road repairs and building of bridges where Government was unable to provide such services. Several communities had built health centres, schools, churches and engaged in many development activities with minimal Government support. It was customary to find community projects where Government made it mandatory for communities to pay counterpart funding to promote ownership. *“They are involved in building a classroom block and they provided land for secondary school project. They also built public toilets.”*In depth interview, Community Leader, Efat Mbat, Bakassi LGA.

“The women association take it upon themselves to clean the entire community every 15th of each month, while the men take care of the roads by making them accessible.” In depth interview, Community Leader, Ibalebo, Abi LGA. “He said that if the paramount ruler of Efut, Muri Efiong Mbukpa, is informed, he will motivate community members to participate. Others desire that the visitation by the team will yield positive results and bear fruits.” FGD, adult male in Archibong Ika, Calabar Municipality.

**Challenges, opportunities and synergies in the delivery of PHC interventions:** Challenges like inadequacy in manpower and commodities were identified at the three levels of PHC. Opportunities abound such as the existence of donor agencies and willingness of communities to participate. Synergies through intersectoral linkages with other service can be harnessed to increase joint usage of resources to improve services including PHC.

## Discussion

The health of a community is always a priority for governments and PHC is a veritable tool for achieving essential care for all. Community participation remains crucial to the success of PHC interventions. This study has demonstrated that the communities acknowledge these facts and yearn for greater involvement in the overall planning of health interventions. This is likely to enhance needs-based and demand-driven provision of health services while promoting sustainability and ownership [5]. Community participation in PHC enhances its affordability compared to conventional, vertical programmes, as recurrent costs become more affordable [6].

In this study, the current national health policy was perceived to be comprehensive as it incorporated eye, mental and dental care into PHC services. However, there appears to be a gap between policy formation and implementation as only immunization services are provided in a comprehensive manner at PHC level. There is a need to strengthen the PHC system to improve implementation of all the elements while ensuring an even distribution of resources. The lack of equitable distribution of resources for PHC was earlier identified as a drawback of the existing health system. Greater transparency and accountability is also required as breakdown of money released for PHC activities was not readily available. Also, PHC staff need to be constantly motivated to perform by provision of training opportunities and promotion. The provision of free drugs for women and children remains a welcome development but it has caused the men to feel neglected. The perception that PHC services are not affordable and beyond the reach of the average community member needs to be addressed. Efforts should be geared towards providing an affordable and holistic package that is accessible by all within the community. The altruistic goal of providing essential health care is made achievable by the presence of NGOs, NGDOs and CBOs to collaborate at state, local government and community levels. These collaborations can be further exploited to yield greater results.

This research has also highlighted the enormous influence that women wield within the communities. This is in keeping with findings of similar studies carried out in Nigeria [7, 8]. This influence should be harnessed by bringing women into the mainstream of PHC services. The channels for achieving this abound within the community. They include the churches, social clubs and community development associations. By putting women at the forefront of PHC activities, a greater impact is likely to be achieved.

## Conclusion

Community directed interventions remain the gold standard for health programmes at the local level. The African Programme for Ochocerciasis Control (APOC) adopted the strategy of Community-Directed Treatment with Ivermectin (CDTi) in the mid-1990s with great success. This strategy was instrumental to the tremendous success achieved in the control and elimination of Onchocerciasis [9, 10]. This community participation can serve as the fulcrum for the revitalization of PHC services within Cross River State and Nigeria. The present research demonstrates a commitment by the community to improving healthcare via CDI. Therefore, CDI should be promoted to strengthen PHC in collaboration with other development partners.
